# MAL Is a Regulator of the Recruitment of Myelin Protein PLP to Membrane Microdomains

**DOI:** 10.1371/journal.pone.0155317

**Published:** 2016-05-12

**Authors:** Marjolein Bijlard, Jenny C. de Jonge, Bert Klunder, Anita Nomden, Dick Hoekstra, Wia Baron

**Affiliations:** Department of Cell Biology, University of Groningen, University Medical Center Groningen, Groningen, the Netherlands; Aix Marseille University, FRANCE

## Abstract

In oligodendrocytes (OLGs), an indirect, transcytotic pathway is mediating transport of *de novo* synthesized PLP, a major myelin specific protein, from the apical-like plasma membrane to the specialized basolateral-like myelin membrane to prevent its premature compaction. MAL is a well-known regulator of polarized trafficking in epithelial cells, and given its presence in OLGs it was therefore of interest to investigate whether MAL played a similar role in PLP transport in OLGs, taking into account its timely expression in these cells. Our data revealed that premature expression of mCherry-MAL in oligodendrocyte progenitor cells interfered with terminal OLG differentiation, although myelin membrane formation *per se* was not impaired. In fact, also PLP transport to myelin membranes via the cell body plasma membrane was unaffected. However, the typical shift of PLP from TX-100-insoluble membrane domains to CHAPS-resistant, but TX-100-soluble membrane domains, seen in the absence of MAL expression, is substantially reduced upon expression of the MAL protein. Interestingly, not only *in vitro*, but also in developing brain a strongly diminished shift from TX-100 resistant to TX-100 soluble domains was observed. Consistently, the MAL-expression mediated annihilation of the typical membrane microdomain shift of PLP is also reflected by a loss of the characteristic surface expression profile of conformation-sensitive anti-PLP antibodies. Hence, these findings suggest that MAL is not involved in vesicular PLP trafficking to either the plasma membrane and/or the myelin membrane as such. Rather, we propose that MAL may regulate PLP’s distribution into distinct membrane microdomains that allow for lateral diffusion of PLP, directly from the plasma membrane to the myelin membrane once the myelin sheath has been assembled.

## Introduction

Oligodendrocytes (OLGs) belong to the glial cell population of the central nervous system (CNS) and are responsible for the production of myelin. Myelin constitutes a multilamellar membrane organization that ensheaths and insulates the axon, thereby facilitating saltatory conduction and providing axonal protection (as reviewed by [1]). Neurological disorders such as multiple sclerosis (MS) are initiated by disruption and degradation of myelin, including failure of OLG differentiation and proper *de novo* biogenesis of myelin sheaths for regeneration. Clearly, a detailed understanding of extra- and intracellular molecular mechanisms that promote myelination, including the biosynthesis and transport of specific myelin membrane components to the myelin sheath, will be instrumental in efforts to develop an effective therapy for such a disease.

The myelin membrane is continuous with the plasma membrane of the OLG, but their composition and underlying mechanisms involved in delivery of their membrane constituents, differ significantly [2–6]. Hence, analogous to epithelial cells and neurons, these myelin-producing cells can be considered as polarized cells. Indeed, previously we have shown that the t-SNAREs syntaxins 3 and 4, which are asymmetrically distributed in (polarized) epithelial cells [7,8], are similarly asymmetrically distributed in OLGs, syntaxin 3 being enriched at the plasma membrane of the cell body, whereas syntaxin 4 localizes towards the myelin membrane [4,9]. Moreover, a transcytotic transport mechanism appears to operate between cell body plasma membrane and myelin membrane in cultured OLGs [10,11]. In fact, the major myelin-specific multispanning proteolipid protein (PLP), comprising 17% of the total fraction of myelin [12] and mediating membrane compaction via clustering of extracellular leaflets [13,14], reaches its final destination via this indirect, transcytotic pathway [11]. Thus, prior to reaching the myelin membrane, PLP is first transported to the apical-like cell body plasma membrane from where the protein is internalized and stored in an endosomal compartment [11,15–18]. From this storage site, the protein is subsequently transported towards the basolateral-like myelin membrane, a process that occurs under neuronal control [19]. Interestingly, along this transcytotic transport pathway, initial transport of *de novo* synthesized PLP from Golgi to plasma membrane relies on its integration in membrane microdomains, characterized by PLP’s resistance to solubilization by Triton X-100 (TX-100) detergent. TX-100 insolubility appears a transient phenomenon, since subsequent to arrival at the cell body plasma membrane the protein segregates in a sulfatide-dependent manner into TX-100 soluble, but CHAPS-insoluble domains [11,20]. Intriguingly, this shift between domains is accompanied by changes in the conformation of the second extracellular loop of PLP and/or its state of oligomerization. Instrumental in transcytotic PLP transport are, among others, the t-SNARE syntaxin 3, which mediates PLP’s insertion into the cell body plasma membrane [11], and myelin and lymphocyte protein 2 (MAL2), which is known to interact with PLP in an ‘apical recycling endosome’-like compartment upon its internalization from the plasma membrane [10].

In the CNS, another member of the MAL family, MAL, is upregulated in OLGs during the period of active myelination, i.e., 3–5 days after the onset of PLP expression [21–23]. Interestingly, and in contrast to MAL2, MAL is a regulator of *direct* apical sorting and delivery in epithelial cells [24–26]. Therefore, MAL may interfere with PLP trafficking, as the protein is known to tightly associate with galactosylceramide (GalC) and sulfatide, both lipids being relevant to PLP’s localization in distinct membrane microdomains [11,20,27]. At steady state, MAL is predominantly localized in compact myelin and colocalizes with PLP and MBP [28]. It has been suggested to be involved in stabilization and maintenance of membrane domains in myelin, while a role in the maintenance of axon-glia interactions has also been proposed [29,30]. Furthermore, in MAL-deficient mice the expression levels of MAG, MBP, and NF155 in myelin and myelin-derived membrane microdomains were reduced [29]. These observations might thus hint at a role for MAL in the delivery efficiency of myelin-specific proteins at the level of sorting and/or trafficking of myelin-directed transport vesicles.

Here, we have addressed the question whether expression of MAL interferes with PLP trafficking. To this end, we expressed MAL in oligodendrocyte progenitor cells (OPCs), PLP-expressing OLN-93 and polarized HepG2 cells, and studied the expression, trafficking, localization and lateral membrane distribution of PLP. Our data indicate that vesicular trafficking of *de novo* synthesized PLP to the cell body plasma membrane is not affected by MAL, but rather that the protein interferes with PLP’s partitioning in TX-100-soluble, CHAPS-insoluble membrane microdomains. We propose that when myelin membrane assembly progresses and/or during maintenance, when a compact myelin membrane organization has been realized, this mechanism would preclude extensive PLP internalization via CHAPS-resistant membrane domains, and rather, allows for ‘direct’ lateral segregation of PLP from the plasma membrane towards the myelin membrane.

## Materials and Methods

### Constructs

PLP-eGFP (pEGFP-N1-PLP) and mCherry-MAL (N-terminally tagged) were kind gifts of Dr. Niels Hellings (Hasselt University, Belgium), and Dr. Miguel Alonso (Universidad Automona de Madrid, Spain), respectively. The mCherry-MAL insert was excised with AgeI and Kpn1 from the pmCherry-C1 plasmid and ‘blunt-end’ ligated into the EcoRV-site of the pENTRA1A plasmid. Inserts from pENTR1A were recombined into pLenti-CMV-Pur (Addgene #17452) or pLenti-CMV/TO-Hygro (Addgene #17291) using Gateway® LR Clonase II Enzyme mix (Life Technologies) according to manufacturer’s instructions. The orientation and the integrity of the obtained constructs were confirmed by DNA sequencing.

### Cell culture

#### Primary oligodendrocytes

Pregnant Wistar rats were purchased from Harlan (Netherlands). Animal protocols were evaluated and approved by the Institutional Animal Care and Use Committee of the University of Groningen (Netherlands). Primary cultures of OLGs were prepared from forebrains of 1–2 day old Wistar rats using a shake-off procedure as described previously [31,32]. OPCs were plated in SATO medium supplemented with the growth factors FGF-2 (10 ng/ml, Peprotech, London, UK) and PDGF-AA (10 ng/ml, Peprotech) on poly-L-lysine (PLL, 5 μg/ml, Sigma, St. Louis, MO)-coated 10 cm dishes (Nunc, Naperville, IL, 1.0 10^6^ cells/dish) or 13-mm-coverslips (30,000 cells/well), for biochemical and immunocytochemical analysis, respectively. After 2 days (‘OPCs’), differentiation was initiated by switching to SATO medium supplemented with 0.5% fetal calf serum (FCS, Bodinco, Alkmaar, the Netherlands), and cells were grown for 3 days (imOLGs) or 7–10 days (mOLGs).

#### OLN-93 cells

The rat-derived oligodendroglia derived cell line, OLN-93 (a kind gift of Dr. Christiane Richter-Landsberg, University of Oldenburg, Germany, [33]) that express the galactolipids GalC and sulfatide with PLP-eGFP (OLN-PLP-GS, [11]) or without PLP-eGFP (OLN-GS, [27,34]) were cultured in DMEM containing 10% FCS. After transduction of OLN-GS and OLN-PLP-GS cells with mCherry-MAL, transduced cells (OLN-GS-MAL and OLN-PLP-GS-MAL) were selected in 100 μg/ml hygromycin for 5 days. Transfection of OLN-GS-MAL cells with the plasmid pLEGFP-N1-PLP was performed with Lipofectamin 2000 (Invitrogen, Breda, The Netherlands), according to the manufacturer's instructions. OLN-93 cells were plated in 10 cm dishes (200.000 cells/dish) for Western Blot and gradient analysis, or on 13-mm-coverslips (10.000 cells/coverslip) for immunocytochemical analysis and grown for 2–3 days.

#### HepG2 cells

HepG2 cells that stably express PLP-eGFP (HepG2-PLP, [11]) were cultured in DMEM containing 10% FCS. After transduction of HepG2-PLP cells with mCherry-MAL, transduced cells were selected in 400 μg/ml hygromycin for 5 days. HepG2 cells were plated in 10 cm dishes (500.000 cells/dish) for Western Blot and gradient analysis, on 13-mm-coverslips for immunocytochemical analysis (30.000 cells/coverslip), or 40.000 cells in a Labtek chambered coverglass (Nunc # 155383) for live-imaging and analysed after 2 days in culture.

### Rat brain tissue

Brain tissue of Wistar rats was homogenized, using a Wheaton homogenizer, in 1 ml of ice-cold TE buffer [10 mM Tris-HCl, 2 mM EDTA, 0.25 M sucrose and a cocktail of protease inhibitors (Complete Mini, Roche Diagnostics, Mannheim, Germany)] at the indicated age. Samples were stored at -80°C until further biochemical analysis.

### Production of lentiviral particles and transduction

For production of lentiviral particles, the constructs, packaging, and envelope plasmids (pRSV-Rev and pMD.G) were transfected into the HEK293T packaging cell line using calcium phosphate. Two days after transfection, medium was refreshed, and conditioned medium was collected after 24 hrs, filtered (Millipore, 0.45 μm pore size), and either used immediately or stored frozen at -80°C. OLN-93 cells, HepG2 cells, and OPCs (48 hrs before the shake-off procedure) were transduced for 16 hrs with lentiviral particles supplemented with 4 μg/mL hexadimethrine bromide (polybrene; Sigma).

### Detergent extraction and membrane microdomain isolation

Cells were washed with PBS, and harvested by scraping the cells in TNE-lysis buffer (50 mM Tris-HCl, pH 7.5, 150 mM NaCl, 5 mM EDTA, and protease cocktail inhibitors) containing either 1% TX-100 or 20 mM 3-[(3-cholamidopropyl)dimethylammonio]-2-hydroxy-1-propanesulfonate (CHAPS). Tissue homogenates were directly extracted with either detergent. The solution was passed 10 times through a 21-gauge needle and incubated on ice for at least 30 min. The protein content was determined by a Bio-Rad DC Protein Assay (Bio-Rad Laboratories, Hercules, CA), using bovine serum albumin (BSA) as standard. Detergent soluble and insoluble fractions were obtained as described previously [35]. Membrane microdomains were isolated using density gradient centrifugation and a discontinuous OptiPrep gradient. To this end, equal amount of protein (500 μg in 250 μl) of total cell detergent extracts or tissue homogenates were added to 500 μl of 60% OptiPrep (Lucron Bioproducts, Milsbeek, the Netherlands). This 40% OptiPrep solution was overlaid with 30% and 10% OptiPrep. Gradients were centrifuged overnight at 152,000 g (SW55 Beckman, 4°C) and seven gradient fractions were collected from the top (fraction 1) to the bottom (fraction 7), and subjected to Western blot or dot blot analysis.

### Immunocytochemistry

Cells were stained with antibodies for the cell surface lipids GalC and sulfatide (Ranscht-mAb (R-mAb), a kind gift of Dr. Guus Wolswijk, NIN, Amsterdam, The Netherlands, [36]), A2B5 (1:10, a kind gift of Dr. Thijs Lopes-Cardozo, University of Utrecht, The Netherlands), PLP (ET3, 1:50, a kind gift of Dr. Elisabeth Trifilieff, University of Strasbourg, France, [11]), and O10 (1:5, a kind gift of Dr. Evi Albers-Krämer. University of Mainz, Germany, [37]). After blocking non-specific binding with 4% BSA in PBS for 10 min, cells were incubated with primary antibodies for 30 min, washed three times and incubated for 25 min with appropriate FITC- or TRITC-conjugated antibodies (1:50, Jackson ImmunoResearch, West Grove, PA). The cells were fixed with 4% paraformaldehyde (PFA) in PBS for 20 min at room temperature (RT), after which the nuclei were stained with DAPI (1 μg/ml, Sigma). For staining of intracellular antigens, cells were fixed with 4% PFA for 20 min at RT. Fixed cells were either permeabilized with ice cold methanol (MBP) for 10 min and subsequently blocked with 4% BSA in PBS for at least 30 min at RT, or with 0.1% TX-100 (PLP, MRP2, MAL) in 4% BSA in PBS for 30 min. The cells were incubated for 1–2 hrs with primary antibodies directed against MBP (Serotec, Oxford, UK, 1:250), PLP (2D2, 1:5, a kind gift of Dr. Vijay. Kuchroo, Harvard Medical School, Boston, MA, [38]), MAL (6D9, 1:100, a kind gift of Dr. Miguel Alonso, Universidad Automona de Madrid, Spain), and MRP2 (1:300, Axxora, Lörrach, Germany) at RT. The cells were washed with PBS and incubated with FITC-conjugated secondary antibodies supplemented with DAPI for 25 min at RT. The samples were analyzed with an immunofluorescence microscope (Olympus AX70 or Leica DMI 6000 B), or a Leica SP8 AOBS CLSM confocal microscope (Leica Microsystems, Heidelberg, Germany) in combination with Leica Confocal Software. Data were processed using Adobe Photoshop software. OLGs were characterized by morphology, i.e., cells with a typical astrocytic morphology were excluded, and in each experiment at least 300 cells were scored as either antigen-positive or antigen-negative. To compare the percentage of PLP-eGFP-positive bile canalicular (BC) apical lumens between the conditions, images were acquired with a Zeiss AcioObserver Z1 microscope equipped with TissueFAXs (Tissuegnostics, Vienna, Austria). The number of PLP-eGFP-positive BCs of MRP2-positive BCs per mm^2^ was determined by TissueQuest analysis (Tissuegnostics).

### Live cell imaging

For live cell imaging of HepG2-PLP and HepG2-PLP-MAL cells 2 days after plating were used. Cells were imaged every minute for 1 hour at 37°C, 5% CO_2_ in the presence of cycloheximide (50 μg/ml) using a Solamere Nipkow confocal microscope. The direction of PLP-eGFP-containing vesicles was determined in Fiji using the manual tracker plugin (Fabrice Cordelières [39]). To this end, 10 vesicles per cell were tracked for 6 min in 5 cells in each of the three independent experiments. Notably, it is not possible track the vesicles for 1 hour, since the vesicles disappear from the focal plane as a function of time. The direction was scored as towards or away from the BC when a clear direction was apparent.

### Western blot and dotblot analysis

Cells pellets were lysed in TNE-lysis buffer for 30 min on ice. Equal protein amounts (20 μg for cells, 25 μg for tissue) or volume (60 μl for gradients) were mixed with SDS-reducing sample buffer, heated for 5 min at 95°C or 30 min at 37°C (PLP) and subjected to SDS-PAGE (10% or 12.5% SDS-gels) and Western blotting as described previously [32]. For dotblot analysis, equal volumes of the gradient fractions (10 μl) were applied onto nitrocellulose membrane, and when dried subjected to similar immunoblot analysis as for the Western blots. Primary antibodies used were anti-GFP (1:500, Invitrogen, Molecular Probes, Eugene, OR), anti-PLP (4C2, a kind gift of Dr Vijay. Kuchroo, Harvard Medical School, Boston, MA, [38]), anti-MAL (6D9, 1:400, [40]), anti-caveolin (BD Transduction Laboratories, Lexington, KY), or anti-actin antibody (1:1000; mouse monoclonal, Sigma). GM1 was visualized with biotinylated-CTB (Sigma). The signals were detected using the Odyssey Infrared Imaging System (Li-Cor Biosciences, Lincoln, NE) and analysed using Odyssey V3.0 analysis software or Scion Image software (Scion Corp., Frederick, MD). The lateral distribution of PLP(-eGFP), caveolin and GM1 in the cell lines was calculated from the protein’s (infrared) intensity in either fractions 3, 4 and 5 (membrane microdomains) or fractions 6 and 7 (non-membrane microdomains), relative to the total intensity, i.e., measured collectively in all fractions.

### Statistical analysis

Data are expressed as mean ± standard deviation (SD) and were obtained from at least three independent experiments. Statistical analysis was performed using a two-tailed Student’s t-test for comparison between two means or an one sample t-test when compared relative to control. For the latter the control was set to 100% in each independent experiment, and the relative values of the other conditions calculated. In all cases a *p* value of *p*< 0.05 was considered significant.

## Results

### Premature MAL expression in OPCs impairs terminal oligodendrocyte differentiation

**Fig 1:**
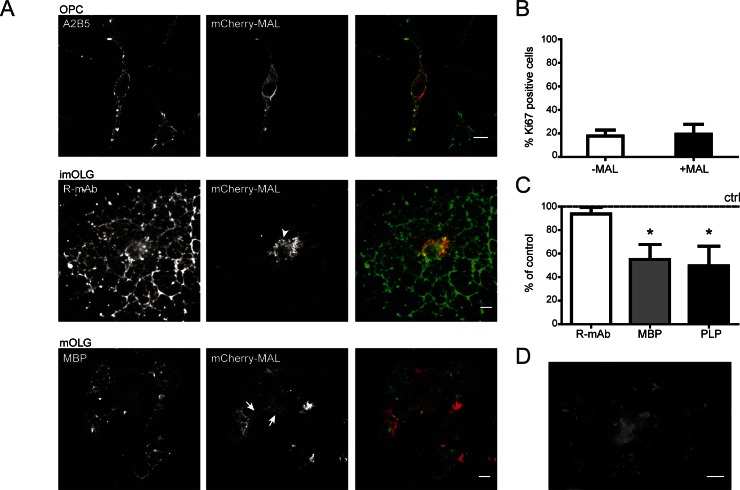
Localization and effect of (mCherry)-MAL on proliferation and differentiation upon expression in oligodendrocyte progenitor cells. mCherry-MAL was lentivirally transduced in oligodendrocyte progenitor cells (OPCs) and cells were allowed to differentiate till immature (imOLGs) and mature (mOLGs) oligodendrocytes. (A) Localization of mCherry-MAL in A2B5-positive OPCs, R-mAb-positive imOLGs, and MBP-positive mOLGs. Representative pictures of at least three independent experiments are shown. (B) The percentage of proliferative OPCs, i.e., Ki67-positive of total mCherry-MAL-positive and mCherry-MAL-negative cells was determined. Each bar represents the mean + SD of three independent experiments. Statistical analysis were performed using the student’s t-test (not significant). (C) The percentage of R-mAb-, MBP-, and PLP-positive mOLGs of total mCherry-MAL-positive and mCherry-MAL-negative cells was determined. In each experiment, the % of MAL-negative cells was set to 100% (horizontal line). The percentage of R-mAb-, MBP- and PLP-positive cells in MAL-negative cells were 80.6.±8.7%, 35.4.±14.7% and 60.0±9.3%, respectively. Each bar represents the mean + SD of three to five independent experiments. Statistical differences with mCherry-MAL-negative cells as assessed with an one sample t-test are shown (*p<0.05). Note that the number of MBP- and PLP-positive cells, but not R-mAb-postive cells, is reduced in cells that express mCherry-MAL. (D) Localization of endogenous MAL in mOLGs as visualized with an anti-MAL antibody (6D9). Scale bars are 10 μm.

The relatively delayed developmental expression of endogenous MAL has hampered progress in understanding its potential function in trafficking of (myelin) proteins in OLGs. Also, in our primary cultures only a few OLGs express endogenous MAL, while the expression levels are too low to detect on Western blot (data not shown). We therefore decided to express exogenous MAL in OPCs, which would enable us to monitor PLP trafficking upon maturation of the cells to OLGs. Accordingly, an N-terminally tagged mCherry-MAL construct was expressed in OPCs by means of lentiviral transduction. Importantly, previous studies have shown that the localization of N-terminus tagged MAL is virtually indistinguishable from that of endogenous MAL, while C-terminus tagged MAL is retained in the endoplasmic reticulum [41]. In polarized MDCK cells, endogenous MAL has been described as an itinerant protein that cycles between the trans-Golgi network and the plasma membrane [42]. To verify whether similar properties apply to mCherry-MAL-expressing cells of the OLG lineage, we first examined its localization during OLG maturation, using stage-specific markers. Upon maturation of OPCs to OLGs, distinct developmental stages can be distinguished. Overall, the cell morphology changes from a bipolar OPC to one that has multiple primary and secondary processes (immature OLGs), while in mature primary OLGs laminar myelin sheets can be distinguished. As shown in Fig 1A, and consistent with observations in MDCK cells, mCherry-MAL was prominently present at the plasma membrane of the cell body and at the perinuclear region in A2B5-positive OPCs. Upon further differentiation to R-mAb-positive immature OLGs, mCherry-MAL increasingly accumulated in vesicular structures primarily localized near the periphery of the cell body (Fig 1A, arrowhead), whereas in MBP-positive sheet forming OLGs, i.e., at the mature OLG stage, mCherrry-MAL was largely present in the perinuclear region and the primary processes (Fig 1A, arrows). As noted above, in mature MAL-expressing OLGs, the localization of mCherry-MAL and endogenous MAL is comparable (Fig 1A *cf* 1D). Furthermore, the morphology of mCherry-MAL expressing OLGs was indistinguishable from that of control OLGs.

To determine whether (premature) expression of MAL affects the development of OLG maturation, we next examined proliferation and differentiation of mCherry-MAL expressing cells, relative to non-expressing cells. Expression of MAL did not affect the proliferation of the cells (Fig 1B). Thus, in the presence of growth factors PDGF-AA and FGF-2, approx. 20% of both MAL-expressing and control OPCs were positive for Ki67, a marker reflecting cell proliferation, while being absent from resting cells. The onset of terminal differentiation is defined by the surface expression of GalC and sulfatide (recognized by the R-mAb), whereas the expression of MBP and PLP prominently occurs in mature, myelin membrane forming OLGs. Therefore, to establish whether mCherry-MAL affects OLG differentiation, the number of R-mAb-, MBP-, and PLP-expressing cells was determined by immunocytochemistry. One week after initiating differentiation, it is apparent that the levels of R-mAb surface expressing cells in control and mCherry-MAL-expressing cells were virtually identical (Fig 1C). In contrast, in mCherry-MAL-expressing OLGs the number of MBP- and PLP-positive cells was reduced by approx. 40%. Microscopical examination of mCherry-MAL-expressing cells (Fig 1A) revealed that the localization of MBP, in cells expressing the protein, as well as the outgrowth of myelin membranes, were seemingly unimpaired. However, more drastic alterations by premature expression of MAL were detected in the processing of the transmembrane protein PLP, as described next. Thus our data reveal that MAL expression does not interfere with proliferation of OPCs, but rather impeded terminal differentiation of the cells.

### Premature MAL expression modulates the lateral plasma membrane distribution of PLP

**Fig 2:**
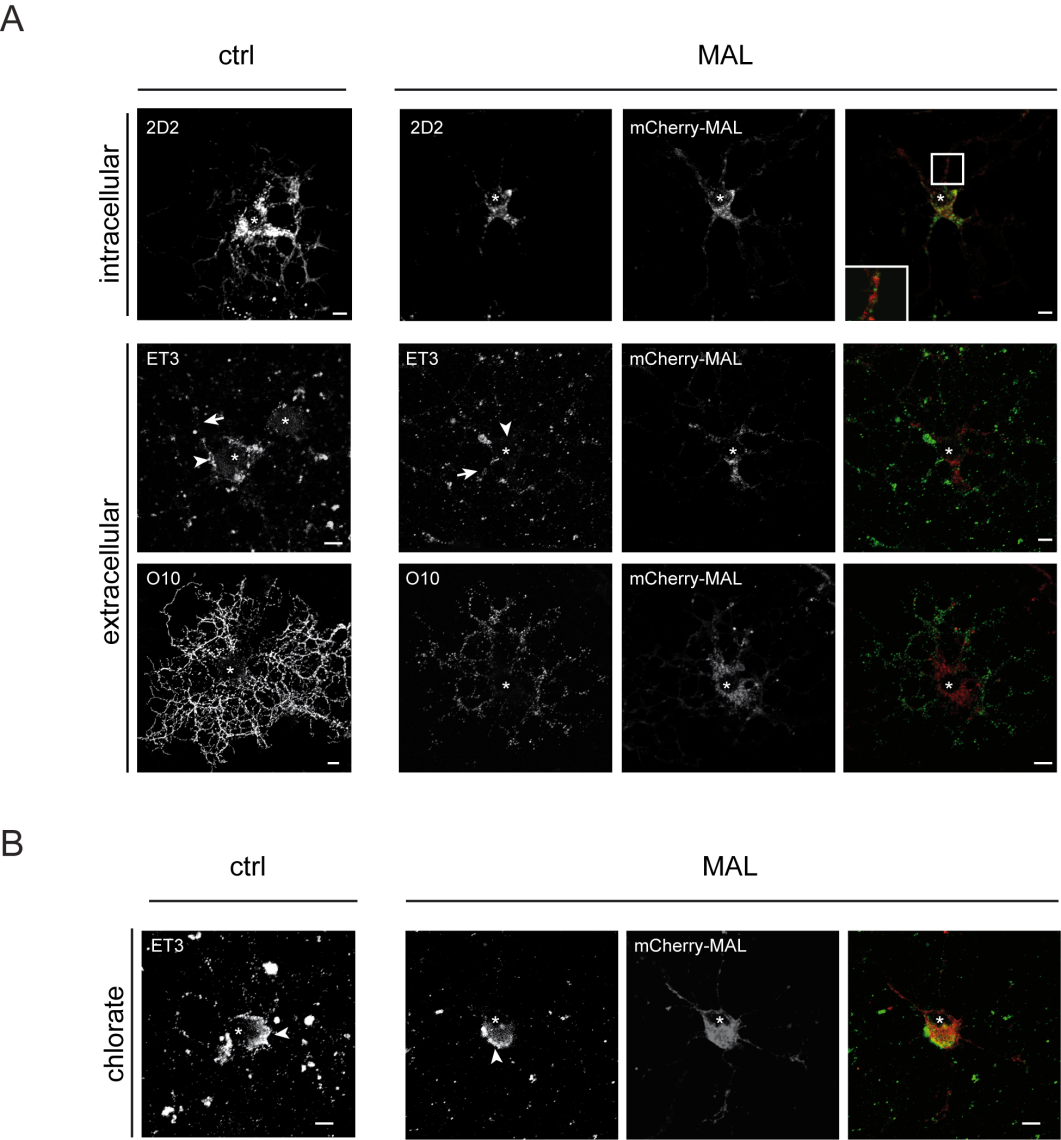
Localization of PLP in mCherry-MAL expressing oligodendrocytes. mCherry-MAL was lentivirally transduced in oligodendrocyte progenitor cells (OPCs) and cells were allowed to differentiate till immature (imOLGs, B) or mature (mOLGs, A) in the absence (A) or presence (B) of sodium chlorate, an inhibitor of sulfatide synthesis and previously shown to block transcytotic PLP transport (chlorate, B, [11]. The intracellular localization of PLP was determined with an anti-PLP antibody (2D2), and the surface localization of PLP was determined by immunostaining using anti-PLP antibodies directed against extracellular epitopes (ET3, O10). Representative pictures of at least three independent experiments are shown. An asterisk (*) indicates the nucleus, arrowheads the cell body plasma membrane, and arrows the primary processes. Scale bars are 10 μm. (A) Note that ET3 primarily binds to the cell body plasma membrane of control mOLGs, whereas its distribution was more dispersed in cells that express mCherry-MAL. Intracellular PLP appears to be more intense in primary processes in MAL-expressing cells rather than the myelin membrane (2D2). (B) Note the similar surface expression of PLP (ET3) at the cell body plasma membrane in control and mCherry-MAL transduced imOLGs upon exposure to chlorate.

As demonstrated above, once terminally differentiated, the overall outgrowth of processes and myelin sheets in MAL-expressing cells in culture, is not impaired based on morphological criteria and MBP expression. Yet, in contrast to MBP, which is transported in mRNA granules and locally expressed in the sheet [43,44], PLP is trafficked in transport vesicles, following biosynthesis at the endoplasmic reticulum. Given the known role of MAL in protein sorting in polarized transport we therefore next investigated whether prematurely expressed MAL might interfere with PLP transport in developing OLGs. First, the localization of PLP was determined by immunofluorescence. As seen in Fig 2A, in mCherry-MAL-expressing cells, PLP seemed to remain concentrated as punctate spots in the cell body and at the beginning of primary processes, while in cells that do not express mCherry-MAL, PLP appeared to be present also within the secondary and tertiary processes (Fig 2A and 2D2). PLP and mCherry-MAL hardly if at all colocalize in the vesicular structures (Fig 2A and 2D2, inset), suggesting that they are not extensively co-transported. PLP is a multispanning membrane protein, and upon its transcytotic transport to the myelin membrane via the cell body plasma membrane, the secondary structure of the second extracellular loop changes as a function of its local environment within the plane of membrane [45]. Thus, although both anti-extracellular PLP antibodies (ET3 and O10) are directed against a conformational epitope in the second extracellular loop, ET3 most prominently binds to PLP when present in TX-100-insoluble membrane microdomains at the cell body plasma membrane, whereas O10 staining is more intense when PLP is localized in the processes and myelin membrane [11,37]. Remarkably, while the ET3 epitope was mainly exposed at the surface of the cell body plasma membrane in control cells, displaying a somewhat clustered appearance, in mCherry-MAL expressing OLGs the ET3 distribution pattern was more laterally diffuse and less confined to the cell body plasma membrane (Fig 2A). The O10 epitope was accessible at the surface, displaying very similar distributions, irrespective of MAL expression, although the intensity of the O10 staining was in most cases reduced in mCherry-MAL-expressing cells (Fig 2A). Hence, despite a substantial intracellular accumulation of PLP in the cell body of MAL-expressing cells, a fraction of PLP is transported to the surface, and subsequently acquires access to the entire OLG surface membrane.

### PLP is transported to the cell body plasma membrane in the presence of MAL

The ET3 staining in particular suggested changes in the overall lateral distribution of PLP in mCherry-MAL-expressing cells. Therefore, the question arises whether the distinction in lateral distribution of PLP derives from different transport pathways operating in mCherry-MAL-expressing cells compared to those seen in control cells. Previously, we have shown that sulfatide *per se* is not necessary for biosynthetic PLP transport to the cell body plasma membrane, while its presence at the plasma membrane is as an essential requirement for subsequent transcytotic PLP transport to the myelin membrane [11]. Therefore, to examine whether a biosynthetic route to the cell body plasma membrane for PLP still operates in MAL-expressing cells, the ET3 surface expression in OPCs that were treated with sodium chlorate, a competitive inhibitor of sulfation, i.e., previously shown to block transcytotic PLP transport, was analyzed next. As shown in Fig 2B, in the absence of sulfatide, surface PLP that harbors the ET3 epitope, remains predominantly localized at the plasma membrane of the cell body in both non-expressing and mCherry-MAL-expressing cells. Thus, these findings show that also in mCherry-MAL-expressing cells PLP is primarily transported to the cell body plasma membrane rather than directly to the myelin-like membranes. Also, the presence of sulfatide is required for subsequent transport to the myelin membrane. Of interest, the seemingly altered localization of mCherry-MAL in the absence of sulfatide (Fig 2B), emphasizes a relatively strong interaction between sulfatide and MAL, as shown previously [28,30]. The interaction between MAL and sulfatide likely affect the distribution of sulfatide in the membrane, which may alter the lateral distribution and surface exposure of the ET3 epitope of PLP in MAL-expressing cells (Fig 2). To analyze whether the observed alteration in surface staining of PLP was related to the protein’s integration in different membrane (micro)domains upon expression of MAL, we next examined the effect of MAL expression on PLP surface expression and lateral membrane distribution *after* PLP is expressed, which more closely mimics the *in vivo* situation. Given the vulnerability of primary terminally differentiated mature OLGs upon lentiviral expression, we were not able to apply lentiviral expression of MAL in these cells after the onset of PLP expression. Therefore, we made use of the oligodendroglial cell line OLN-93. OLN-93 cells do not express MAL and PLP (S1 Fig), allowing for selective exogenous expression of MAL before and after PLP expression.

### MAL modifies the lateral membrane localization of PLP in OLN-PLP-GS cells

**Fig 3:**
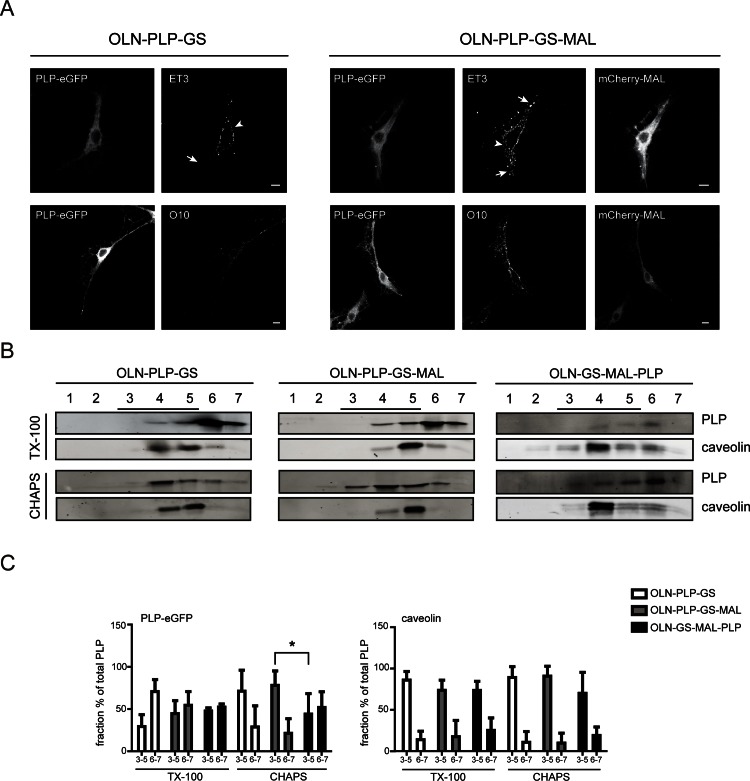
Localization and membrane microdomain association of PLP in OLN-PLP-GS and OLN-PLP-GS-MAL cells. (A) Localization of PLP in OLN-cells in the presence (OLN-PLP-GS-MAL) and absence of MAL (OLN-PLP-GS). MAL is expressed after PLP-eGFP. ET3 and O10 recognize distinct extracellular PLP epitopes. Representative pictures of at least three independent experiments are shown. The PLP-eGFP expression in the cytoplasm marks the morphology of the cells, including the processes. Scale bars are 10 μm. Note that upon expression of MAL the ET3 PLP surface epitope is exposed along the entire membrane (arrows), while being restricted to the cell body plasma membrane in the absence of MAL (arrowheads). (B,C) OLN-PLP-GS (‘without MAL’), OLN-PLP-GS-MAL (‘MAL after PLP’) and OLN-GS-MAL-PLP (‘MAL before PLP’) cells were extracted with TX-100 (1%) or CHAPS (20 mM) at 4°C and subjected to OptiPrep density centrifugation. PLP-eGFP (anti-GFP) and caveolin were visualized by Western blot. Representative blots of two to four independent experiments are shown in B, quantitative analyses in C. The protein percentage of each fraction was calculated by dividing the protein percentage in that fraction by total protein expression. Bar graphs of the pooled fraction percentage of (membrane microdomain, ‘raft’) fractions 3–5 and (‘non-raft’) fractions 6 and 7 are shown. Statistical differences with OLN-PLP-GS cells as assessed with a student’s *t*-test are shown (* p<0.05). Note that upon premature expression of MAL, i.e., prior to transient expression of PLP (OLN-GS-MAL-PLP) the characteristic distribution of PLP in CHAPS-insoluble (membrane microdomain) fractions is reduced, while the lateral distribution of the membrane microdomain marker caveolin is not altered.

To further clarify the role of MAL in PLP transport and its lateral membrane distribution, we made use of our previously established OLN-93-derived cell line OLN-PLP-GS [11]. In contrast to parental OLN-93 cells, OLN-PLP-GS cells express GalC and sulfatide, as well as PLP-eGFP. In these cells PLP-eGFP transport to the plasma membrane displays similar features as in primary OLGs. Thus, in line with the observations in primary OLGs, in OLN-PLP-GS cells, the ET3 epitope is mainly exposed at the cell body, while the O10 epitope emerges at the processes ([11], Fig 3A). In addition, at steady state conditions PLP mainly resides in CHAPS-resistant, TX-100 soluble membrane microdomains [11], Fig 3B). Notably, the expression levels of either PLP or MAL do not change when the proteins are expressed together (S1 Fig). When MAL is lentivirally transduced in OLN-PLP-GS cells (OLN-PLP-GS-MAL), i.e., expressed after PLP expression as occurs during development, the characteristic staining of antibody ET3, almost exclusively restricted to the cell body plasma membrane in the absence of MAL (Fig 3A, arrowhead) becomes severely perturbed. Thus, a pronounced staining of the processes is observed (Fig 3A, arrow), very similar as occurs in primary OLGs upon (premature) MAL expression (Fig 2A, arrow). By contrast, the lateral distribution of the O10 epitope remains largely unaltered. As noted, the exposure of the ET3 and O10 epitopes reflects differences in PLP’s partitioning in different membrane microenvironments [11,45]. Specifically, following biosynthesis, PLP transiently resides in TX-100-resistant membrane microdomains in plasma membrane-directed transport vesicles. At the plasma membrane, the protein is subsequently segregated into CHAPS-resistant, but TX-100-soluble microdomains prior to its internalization and transport to myelin membranes [11,20]. This dynamic is reflected by a membrane-microdomain dependent change in the exposure of the conformational epitopes ET3 and O10, respectively [11,45]. To analyze whether the altered lateral surface distribution of PLP in MAL-expressing cells, as particularly reflected by a change in the lateral distribution of the ET3 epitope, was accompanied by an altered partitioning into membrane microdomains, the detergent (in)solubility of PLP was determined upon extraction with CHAPS or TX-100. Membrane microdomains were isolated via density gradient centrifugation, and PLP was visualized by Western blot. In the gradients, fractions 3 to 5 are considered to represent membrane microdomain (raft) fractions, as reflected by a relative enrichment of the membrane microdomain marker caveolin-1 in these fractions, whereas fractions 6 and 7 represent non-raft fractions (Fig 3B). In MAL-expressing cells, a slight redistribution of PLP to TX-100-insoluble fractions was observed, as compared to non-expressing cells (Fig 3B and 3C 29.3.±14.2% in OLN-PLP-GS vs 44.6±15.3% in OLN-PLP-GS-MAL cells and 47.7.±3.7% in OLN-GS-MAL-PLP cells), while the distribution of caveolin-1 remained similar. In OLN-PLP-GS and OLN-PLP-GS-MAL cells, the major fraction of PLP was CHAPS-resistant (71.1±24.9% in OLN-PLP-GS and 78.1±17.1% in OLN-PLP-GS-MAL cells). In contrast, when MAL was expressed *before* PLP (OLN-GS-MAL-PLP cells), the incorporation of PLP in CHAPS-insoluble membrane microdomains was significantly reduced (44.1±24.5% in OLN-GS-MAL-PLP cells). Since OLN-93 cells do not produce proper myelin sheets and hence, cannot be regarded as genuinely polarized cells, we next investigated the effect of MAL on PLP’s localization in a well-characterized polarized cell model.

### MAL modifies the lateral membrane localization of PLP in HepG2 cells

**Fig 4:**
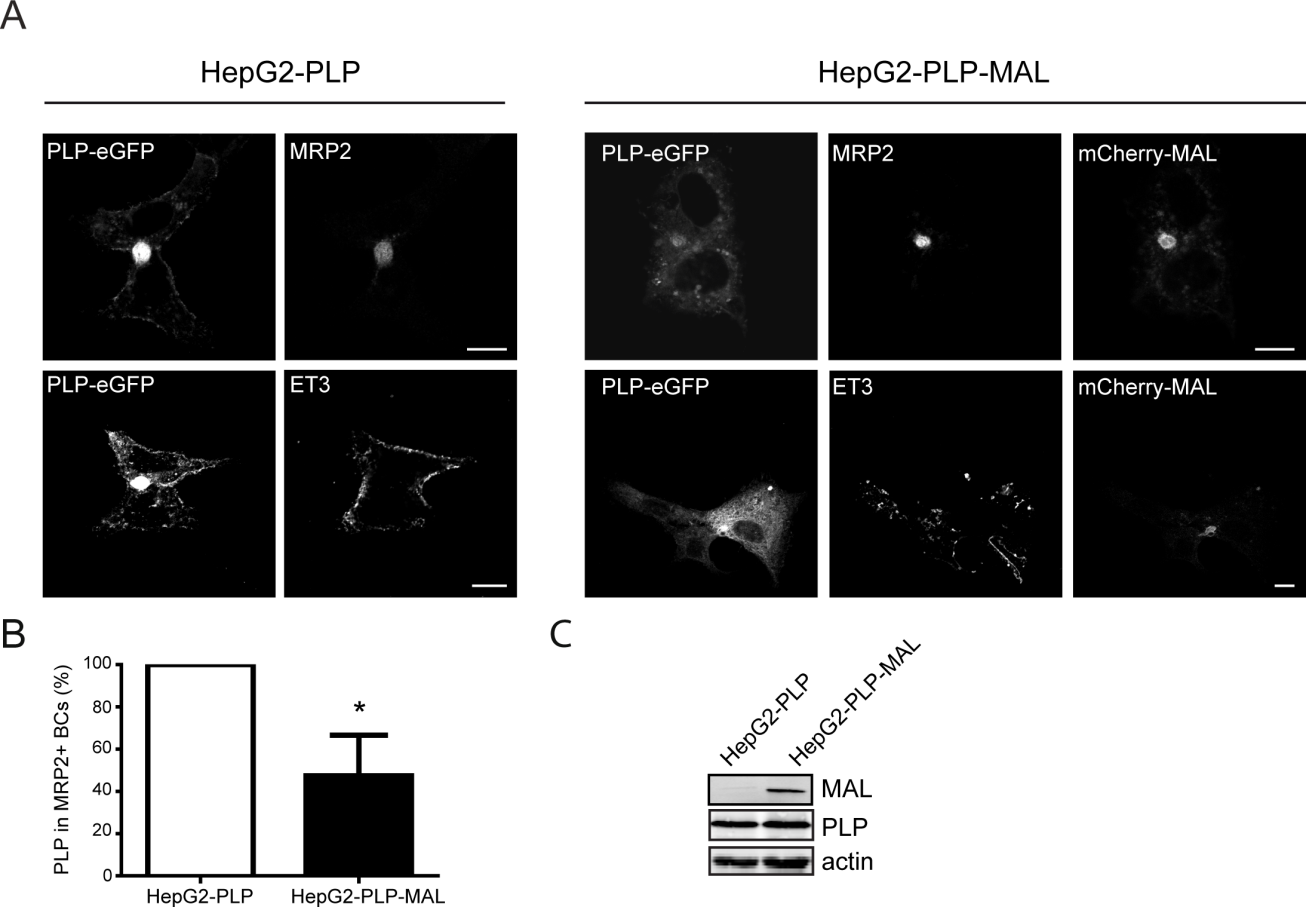
Localization of PLP in HepG2-PLP and HepG2-PLP-MAL cells. (A) Localization of PLP-eGFP in HepG2-cells in the presence (HepG2-PLP-MAL) and absence of MAL (HepG2-PLP). MAL is expressed after PLP-eGFP. MRP2 is an apical marker, ET3 recognizes an extracellular PLP epitope. Representative pictures of at least three independent experiments are shown. Scale bars are 10 μm. Note that upon expression of MAL the expression of PLP-eGFP at the apical surface seems to be reduced; more intracellular structures appear, while still present at the basolateral membrane surface. (B) Quantitative analysis of the number of PLP-positive of total MRP2-positive bile canaliculi (BCs, apical surface) in HepG2-PLP and HepG2-PLP-MAL cells. Each bar represents the mean + SD of three independent experiments. In each experiment, the % of PLP-positive of total MRP2-positive BCs was set to 100%. In HepG2-PLP cells 41.0 ± 9.4% of the MRP2-postitive BCs were PLP-positive. Statistical differences with HepG2-PLP cells as assessed with a one sample *t*-test are shown (* p< 0.05). Note that the number of PLP-positive BCs is reduced upon expression of MAL. (C) Western blot analysis of the expression levels of PLP (anti-GFP) and MAL (anti-MAL antibody, 6D9) in HepG2-PLP and HepG2-PLP-MAL cells. Note that PLP expression is similar in the presence or absence of MAL.

**Fig 5:**
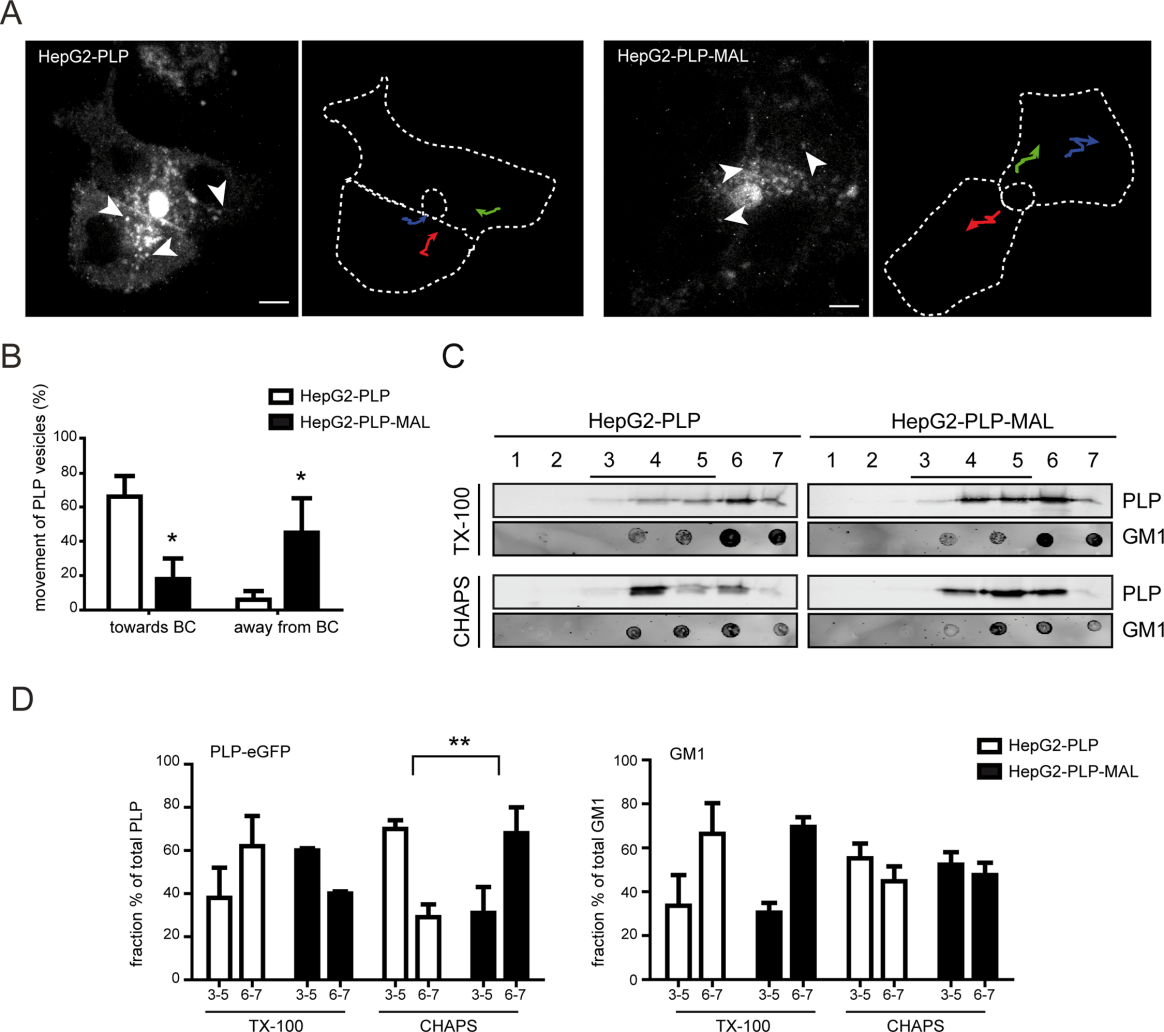
Live cell imaging and membrane microdomain association of PLP-eGFP in HepG2-PLP and HepG2-PLP-MAL cells. (A,B) The movement of PLP-eGFP-containing vesicles was recorded in live HepG2-PLP and HepG2-PLP-MAL cells in the presence of cycloheximide for one hour at 37°C and 5% CO_2_. Images were acquired every minute. The direction of 10 PLP-eGFP-containing vesicles in 5 cells was tracked for 6 min in Fiji using the manual tracker plugin, and scored as towards or away from the BC when a clear direction was apparent. Representative tracks are shown in A (the tracks of 3 vesicles in each cell are indicated, see S1 and S2 Movies for respectively HepG2-PLP and HepG2-PLP-MAL cells), quantitative analysis of three independent experiments in B. Each bar represents the mean + SD. Statistical differences as assessed with a student’s *t*-test are shown (* p< 0.05). Scale bar is 10 μm. (C,D) HepG2-PLP and HepG2-PLP-MAL cells were extracted with TX-100 (1%) or CHAPS (20 mM) at 4°C and subjected to OptiPrep density centrifugation. PLP-eGFP (anti-GFP) and GM1 (CTB) were visualized by Western and dot blotting, respectively. Representative blots are shown in C, quantitative analyses of three independent experiments in D. The protein/lipid percentage of each fraction was calculated by the dividing the protein/lipid percentage in that fraction by total protein/lipid expression. Bar graphs of the pooled fraction percentage of (membrane microdomain, ‘raft’) fractions 3–5 and (‘non-raft’) fractions 6 and 7 are shown. Statistical differences with HepG2-PLP cells as assessed with a student’s t-test are shown (* p<0.05). Note that upon expression of MAL the characteristic distribution of PLP in CHAPS-insoluble (raft), TX-100-insoluble (non-raft) fractions is lost, while the lateral distribution of GM1, a membrane microdomain marker, is not altered.

The hepatic cell line HepG2 may serve as a valuable model to study PLP transport. Polarized HepG2 cells have functional apical and basolateral membrane domains that are separated by tight junctions. In these cells, newly synthesized surface membrane proteins are either transported via direct pathways or via transcytosis to their surface destination, i.e., either the basolateral or apical membrane. Previously, we have shown that in polarized HepG2 cells, PLP-eGFP, when expressed in these cells, localizes to both the apical domain, i.e., the bile canalicular (BC) membrane in liver cells, and the basolateral domain ([11], Fig 4A). Furthermore, at steady state conditions, PLP-eGFP partitions in membrane microdomains, displaying very similar detergent resistance properties as those observed in primary OLGs ([11], see below). Moreover, sulfatide levels are increased in PLP-expressing HepG2 cells [11], while GalC is actively transported, together with sphingomyelin, along a reversed transcytotic pathway (apical to basolateral surface, [46]). This would be consistent with presumed mechanistic features in polarized transcytotic transport of PLP in OLGs, in which transport from the intracellular storage endosomes to the basolateral-like myelin sheet similarly depends on GalC [20]. Moreover, HepG2 cells lack endogenous MAL ([26], Fig 4C), making these cells an ideal model to study the effect of MAL on PLP transport and lateral membrane distribution. As shown in Fig 4A, in control, MAL-devoid HepG2 cells, PLP-eGFP is primarily present at both the apical and basolateral membrane, while only a minor fraction localizes to intracellular structures. In MAL-expressing HepG2 cells, a dramatic shift in PLP distribution is apparent, i.e., apical membrane localized PLP is reduced and, instead, a more prominent intracellular accumulation is apparent. Interestingly, MAL was mainly present at the apical BC membrane, while a minor fraction resided in intracellular vesicular structures. As observed in primary OLGs, MAL does not significantly colocalize with PLP in the intracellular structures. Following the expression of MAL, the number of double stained BC’s, containing both PLP and the apical marker MRP2 was however markedly reduced (Fig 4B, approx. 50%). The localization of MRP2, similarly as PLP a multispanning membrane protein, was not visually affected upon MAL expression, indicating that MAL selectively altered the distribution of PLP. In HepG2 cells, antibody ET3 recognizes extracellular PLP-eGFP at the basolateral surface ([11], Fig 4A), while the O10 epitope is not exposed [11]. Upon MAL expression, a fraction of PLP still localized at the basolateral surface, as visualized with antibody ET3 (Fig 4A, ET3). In fact, vesicle tracking experiments, in which vesicles were carefully monitored that had a clear directional movement towards or away from the BC surface, revealed that in MAL-devoid, control HepG2 cells, PLP-containing vesicular structures appeared to move more towards the BC, while in MAL-expressing HepG2 cells, the vesicles moved away from the BC (Fig 5A and 5B, S1 and S2 Movies). This suggests increased trafficking towards the basolateral membrane. To analyze whether this alteration of PLP transport was accompanied by an altered lateral membrane distribution of the protein, as reflected by differences in detergent (in)solubility, the detergent (in)solubility of PLP was determined upon extraction with CHAPS or TX-100. Expression of MAL altered the relative overall partitioning of PLP in detergent soluble versus insoluble membrane microdomains (Fig 5C and 5D). Thus, following extraction with CHAPS in MAL-devoid control HepG2 cells, 69.7±4.4% of the PLP-eGFP fraction was present in CHAPS-resistant membrane microdomains (fractions 3–5), while only 31.5±12.2% of the PLP-eGFP fraction was present in these membrane microdomains in MAL-expressing HepG2 cells (Fig 5C and 5D). An opposite observation was made in case of analysis of TX-100-resistant membrane microdomains, i.e, PLP-eGFP showed some tendency to segregate into TX-100-resistant membrane microdomains when MAL is expressed (Fig 5C and 5D, 37.8±14.3% in HepG2-PLP vs 60.4±0.9% in HepG2-PLP-MAL cells). As caveolin is not expressed in HepG2 cells, GM1, as visualized on a dotblot with CTB, was used as a marker for membrane microdomains [47]. Incorporation of ganglioside GM1 in CHAPS- or TX-100-insoluble membrane microdomains remained unaltered, i.e., irrespective of the presence of mCherry-MAL. Hence, upon expression of MAL the distinct association of PLP with CHAPS-resistant, TX-100-soluble membrane microdomains is substantially diminished in HepG2 cells. To examine whether a similar lateral membrane redistribution of PLP occurs during myelin maintenance, we next determined PLP’s detergent (in)solubility during rat brain development.

### Increased association of PLP with TX-100-resistant membrane microdomains upon brain development

**Fig 6:**
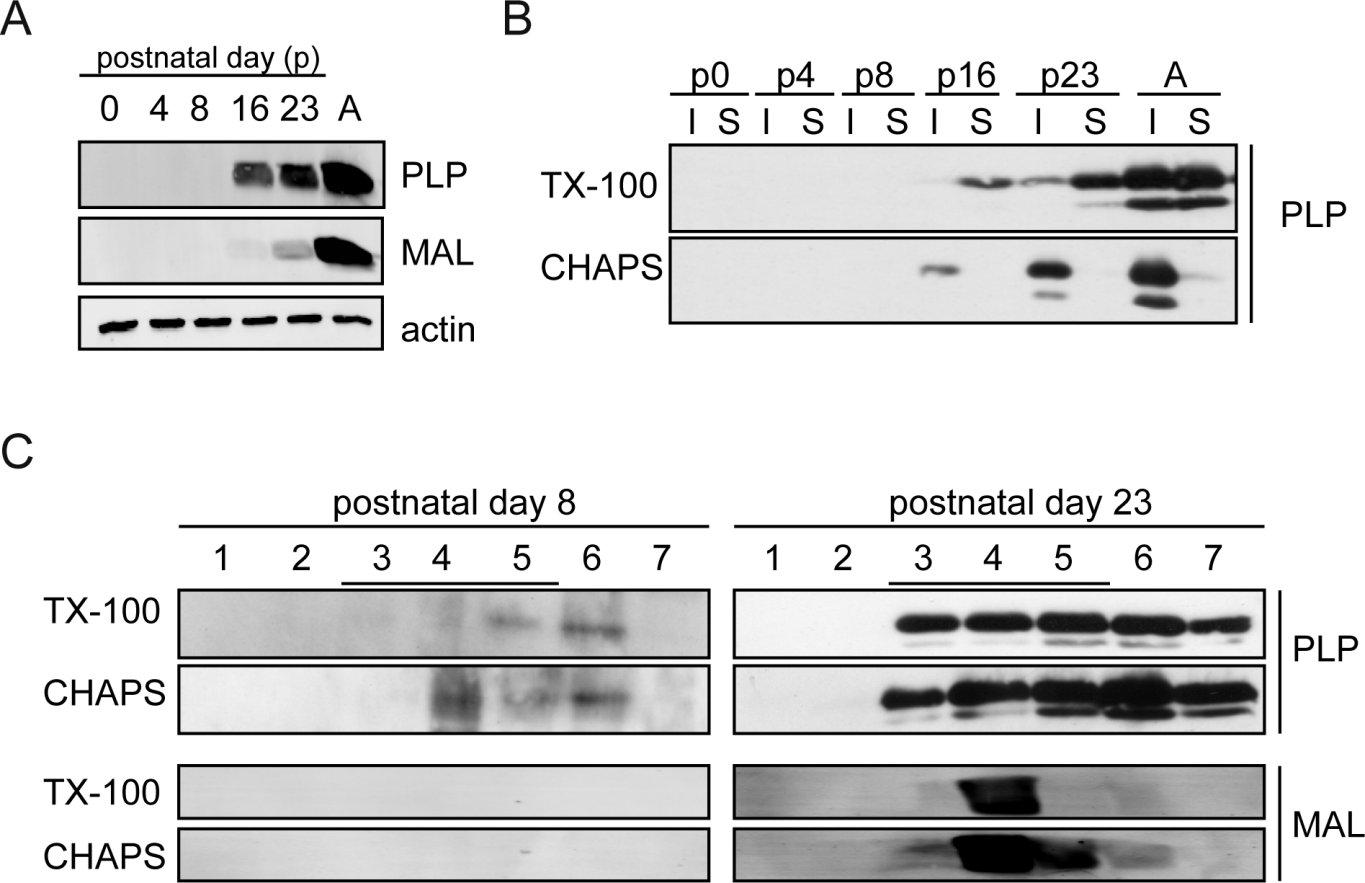
Detergent solubility of PLP during rat brain development. (A) Adult rat brains and brains between postnatal days (p)0-23 were homogenized and analyzed for expression of PLP and MAL by Western blotting. Actin serves as a loading control. Note that PLP is expressed before MAL during rat brain development. (B) After TX-100 (1%) or CHAPS (20 mM) extraction at 4°C and centrifugation, the distribution of PLP in detergent-soluble and -insoluble fraction at the indicated age were analyzed by Western blotting. (C) Brain homogenates of p8 and p23 rats were extracted with TX-100 (1%) or CHAPS (20 mM) at 4°C and subjected to OptiPrep density centrifugation. PLP (4C2 antibody) and MAL (6D9 antibody) were visualized by Western blotting. Detergent-resistant membrane microdomains are present in fractions 3–5. Note that upon brain development, PLP’s insolubility in TX-100 is increased at the time MAL is expressed.

To examine the detergent (in)solubility of PLP during rat brain development, total brain homogenates, isolated from animals of different age, were extracted with CHAPS or TX-100 at 4°C, and soluble and insoluble fractions were separated by centrifugation. As shown in Fig 6A, although detectable at very low levels at postnatal day 8 (p8, Fig 6C), PLP was significantly expressed from postnatal day 16 onwards, i.e., during active myelination, while MAL is detectable at very low levels at postnatal day 16 and clearly expressed at p23. At p16, PLP almost exclusively partitioned in the TX-100 soluble but CHAPS-insoluble fraction (approx. 95%), very similar as observed in cultured OLGs (Fig 6B, [11,20]). At p23, i.e., at the time MAL is expressed (Fig 6A), and even more prominently in homogenates isolated from the adult rat brain, PLP increasingly segregated to the TX-100 insoluble (approx. 5% at p16, 22% at p23, and approx. 49% at adulthood) fraction, while remaining CHAPS-insoluble (approx. 95% at p16, p23 and adulthood). To unequivocally determine the association of PLP with TX-100-resistant membrane microdomains during development, CHAPS and TX-100 extracts of homogenates isolated from p8 and p23 rat brains, i.e., respectively at the onset and the end of myelin biogenesis during rat brain development, were loaded on an Optiprep density gradient and analyzed by Western blot. Whereas in p8 total brain homogenates PLP is mainly recovered in TX-100-soluble (Fig 6C, fractions 6–7, approx. 63%) but CHAPS-insoluble fractions (Fig 6C, fractions 3–5, approx. 62%), in p23 brain homogenates, PLP is more homogenously distributed and localized to both TX-100-insoluble (Fig 6C, fractions 3–5, approx. 61%) and CHAPS-insoluble fractions (Fig 6C, fractions 3–5, approx. 43%). In contrast to PLP, MAL appears to be solely present in membrane microdomains at p23 (Fig 6C, fractions 3–5), further suggesting that MAL and PLP do not always colocalize. Hence, these data indicate that the distinct association of PLP with CHAPS-resistant, TX-100 soluble membrane microdomains is diminishing upon rat brain development.

## Discussion

**Fig 7:**
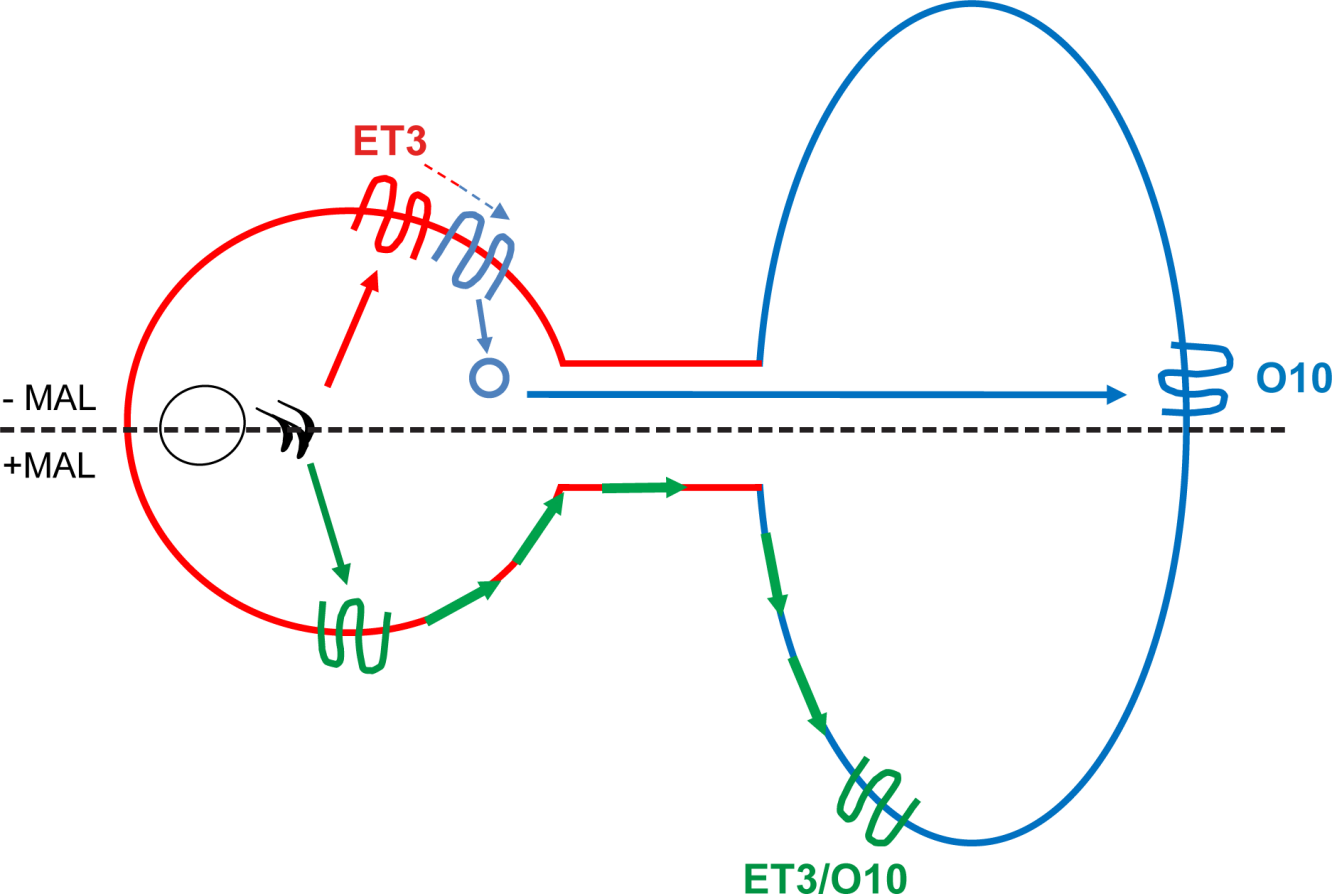
Schematic overview of the trafficking of newly synthesized PLP to myelin membranes in the presence or absence of MAL. In the absence of MAL (upper part), newly synthesized PLP is transported to the myelin membrane via an indirect pathway, involving (i) vesicular transport to the apical-like cell body plasma membrane prior to basolateral-like myelin membranes and (ii) temporal accumulation in a late endosomal compartment [11,20]. Along this route, PLP partitions in distinct membrane domains, i.e., TX100-resistant or CHAPS-resistant, and expose different conformational epitopes during respectively biosynthetic (red, ET3) and transcytotic (blue, O10) transport. In the presence of MAL (lower part), the distinct partitioning of PLP in distinct membrane microdomains is lost and both conformational epitopes are exposed along the entire membrane. These MAL-mediated changes allow for lateral diffusion of PLP from the cell body plasma membrane to the myelin membrane (green, ET3/O10).

The aim of the present work was to obtain further insight into molecular mechanisms underlying the regulation of the biogenesis of myelin membranes in (polarized) OLGs, with a focus on trafficking of the major myelin protein PLP in particular. Our data suggest that MAL, a known regulator of polarized trafficking in epithelial cells, does not interfere with vesicular trafficking of PLP, following its biosynthesis, to the (apical) cell body membrane. Rather, the protein appears to control the lateral segregation of PLP into distinct membrane microdomains, presumably by interfering in a competitive manner with PLP’s interaction with the galactosphingolipid sulfatide. We propose that at such conditions, PLP may acquire direct access from the plasma membrane to the myelin membrane via slow lateral diffusion (Fig 7). Given the timely expression of MAL, this feature may be important to avoid increasingly frustrated vesicular transport when myelin compaction advances, and during myelin maintenance when myelin membranes are established and have less contact with the cytosol.

To comprehend the effect of MAL on PLP trafficking and the significance of PLP’s lateral membrane localization, it is relevant to consider the transport itinerary of the protein in OLGs. In a previous study we have shown that after biosynthesis at the endoplasmic reticulum, PLP is transported via the Golgi to the cell body plasma membrane and subsequently to the myelin sheet, thus following a rapid transcytotic route (Fig 7, upper part, [11]) Importantly, initial transport to the plasma membrane relies on PLP’s integrations into membrane microdomains, characterized by a resistance towards solubilization by TX-100. This initial TX-100 insolubility is transient, since after PLP’s departure from the cell body plasma membrane *en route* to the myelin membrane, the protein resides in TX-100-soluble, CHAPS-insoluble membrane microdomains [11,20,48]. During OLG development, MAL becomes expressed following PLP expression [11,20,48]. Indeed, our data highlight the timeliness of MAL expression as its premature expression halts terminal OLG differentiation, reflected by a decreased number of MBP- and PLP-positive cells. This is consistent with observations in Schwann cells, where MAL is expressed at embryonic age day 18 [49]. Once differentiated, MAL expression did not impair overall myelin biogenesis *per se*, given the appearance of MBP-positive myelin sheets in MAL-expressing OLGs. These findings are in agreement with a normal onset of myelin biogenesis in MAL-deficient mice and the observed hypomyelination when MAL is overexpressed under its own promotor [29,30]. Also, premature expression of MAL does not seemingly interfere with PLP transport to the cell body plasma membrane, following biosynthesis, and at this condition, the protein does acquire access to the myelin membrane.

Our data further reveal that the presence of MAL interfered with the conformation of PLP. As shown previously, when transported along the biosynthetic and transcytotic pathway, PLP undergoes conformational changes, which are instrumental in its overall transport to the myelin membrane [11]. Specifically, the protein adopts a different conformation after reaching the cell body plasma membrane, prior to its transport to the myelin membrane (Fig 7). However, following expression of MAL in OLGs (premature) and OLN-93 cells (after PLP), this conformation-dependent difference in the localization of PLP was no longer apparent. Thus, in MAL-expressing OLGs, both conformation-dependent epitopes appear to be evenly distributed over primary processes and myelin membrane. Since the ability of PLP to adopt a specific conformation appears to be related to its partitioning in different membrane microenvironments [11,45], the less strict conformation-dependent localization upon expression of MAL is likely a reflection of a change in its membrane microenvironment. Indeed, the characteristic lateral membrane distribution of PLP in CHAPS-insoluble, TX-100-soluble membrane microdomains is lost in OLN-93 and HepG2 cells upon expression of MAL, as reflected by an increased TX-100 insolubility and a tendency towards decreased CHAPS insolubility. Likely, this property of PLP is already acquired in the Golgi and not at the plasma membrane, since in WIF-B cells, MAL expression induced the formation of biosynthetic, cholesterol and glycosphingolipid-enriched domains in the Golgi [26]. Of interest in this regard, analysis of MAL-deficient mice revealed that MAL is also required for the proper association of the oligodendroglial paranodal protein NF155 with membrane microdomains [29]. This further indicates that MAL may interfere with membrane microdomain formation *per se* and/or the partitioning of molecular cargo in such domains. Importantly, MAL and PLP do not co-localize in vesicular structures, and are likely not transported together to myelin membranes, indicating that MAL interferes with membrane domain formation by indirect means rather than by a direct interaction. To what extent do the data as presented bear physiological relevance? Parallel CHAPS and TX-100 extractions on whole brain homogenates upon rat brain development, i.e., containing cells, vesicles and myelin, show that the characteristic distribution of PLP in CHAPS-insoluble, TX-100-soluble membrane domains as evidenced in cultured cells, was similarly observed at the onset of myelin biogenesis (age p8). At the time of pronounced MAL expression (age p23), this marked partitioning of PLP in different membrane microdomains is lost, analogous to what occurs in cultured MAL-expressing cells. Consistently, in MAL-deficient mice, PLP is CHAPS-insoluble in adult myelin [29], but without knowledge on its TX-100-(in)solubility, as well as its detergent (in)solubility in total homogenates, conclusions on its role on PLP’s distribution and trafficking in Mal-deficient mice cannot be drawn yet. Also, since there is no consensus yet as to whether PLP resides in CHAPS and/or TX100 insoluble membrane microdomains in vivo. Several studies that analyze the detergent (in)solubility of PLP with either CHAPS or TX-100, show that in purified myelin of adult brains, PLP resides in CHAPS-insoluble, TX-100-soluble membrane microdomains [20,48], while other investigators have shown that PLP is present in TX-100-insoluble membrane microdomains [50–52]. This apparent discrepancy in TX-100 (in)solubility may be related to a difference in the salt concentration of the used solubilisation buffers [52] as well as to the presence or absence of PLP that did not reach myelin membranes yet.

At the molecular level, sulfatide, GalC and cholesterol, i.e., crucial constituents of membrane microdomains are important regulators in transcytotic PLP transport [20,51]. Since MAL may associate with galactolipids [28,53] and facilitate their lateral segregation [54], such interactions may readily lead to an interference with the dynamic partitioning of PLP in the distinct TX-100 and CHAPS detergent-resistant membrane microdomains. Consistently, also in galactolipid-deficient mice and cultured OLGs, the characteristic distribution of PLP in CHAPS-resistant, TX-100-soluble membrane microdomains is abolished [11,20], while PLP transport to myelin membranes still proceeds [55]. Hence, rather than interfering with direct apical delivery of PLP to the cell body plasma membrane, MAL may interfere with subsequent transcytotic transport of PLP. The observed altered distribution of the conformation-dependent epitope, indicate that transport from the cell body plasma membrane to the myelin membrane may have occurred independent of transcytotic transport, i.e., via lateral diffusion (Fig 7, lower part). Alternatively, once deposited at the myelin membrane via transcytotic transport, its molecular environment in the myelin membrane may have (partly) altered the conformation of PLP in MAL-expressing cells in such a way that the ET3 epitope reappears. Our preliminary data show that in MAL-expressing cells PLP-containing vesicles do not significantly colocalize with LAMP-1, a marker of the presumed endocytic storage compartment of PLP *en route* to the myelin membrane [15,19]. This favors the scenario of privileged transport of PLP to the myelin membrane via slow lateral diffusion upon expression of MAL over rapid vesicular transcytotic transport. Consistently, neuronal signals that trigger PLP trafficking to myelin membranes, cause a diminished internalization of PLP by endocytosis [19], which represents an essential step in the transcytotic pathway. Also, a change in PLP transport is observed in PLP-expressing HepG2 cells upon expression of MAL. MAL strongly reduced the localization of PLP at the apical BC membrane and favors transport to the basolateral membrane. Of note, while in OLGs lateral diffusion of PLP may occur between plasma membrane and myelin membrane, since these membrane domains are continuous, the presence of tight junctions, as seen in HepG2 cells, prevents lateral distribution from the apical to basolateral membrane. Whether MAL indeed facilitates lateral diffusion of PLP in OLGs remains to be determined.

Taken together, the present findings show that MAL modulates the recruitment of PLP to membrane microdomains. During development, MAL expression occurs subsequent to PLP expression at a stage where most of the PLP has been synthesized and transported and myelin compaction has already started. At this stage, vesicular trafficking into the tightly packed sheath is presumably severely frustrated. Accordingly, we submit that the MAL-induced alteration in the recruitment of newly synthesized PLP into distinct membrane microdomains, enables for the delivery of PLP into the myelin sheath via direct lateral diffusion between plasma membrane and myelin membrane, rather than by means of vesicular transport [6,56].

## Supporting Information

S1 Fig Expression of MAL and PLP in OLN-cells. The expression of MAL and PLP in OLN-GS, OLN-PLP-GS, OLN-PLP-GS-MAL and OLN-GS-MAL cells were analyzed by Western blotting using anti-PLP (4C2) and anti-MAL (6D9) antibodies. Actin serves as a loading control. Note that OLN-GS do not express endogenous PLP and MAL, while the expression levels of PLP-eGFP and mCherry-MAL were similar in OLN-PLP-GS-MAL as compared to OLN-PLP-GS and OLN-GS-MAL cells respectively.
(TIF)

S1 Movie Live cell imaging of PLP-eGFP in HepG2-PLP cells. The movement of PLP-eGFP-containing vesicles was monitored in live HepG2-PLP cells by time-lapse microscopy. Cells were recorded in the presence of cycloheximide for 1 hour at 37°C and 5% CO_2._ Images were taken every minute. The direction of 3 PLP-eGFP-containing vesicles in HepG2-PLP cells was tracked for 6 min each in Fiji using the manual tracker plugin. The video is played at 3 frames/sec; total time for shown image sequence is 9 min.
(AVI)

S2 Movie Live cell imaging of PLP-eGFP in HepG2-PLP-MAL cells. The movement of PLP-eGFP-containing vesicles was monitored in live HepG2-PLP-MAL cells by time-lapse microscopy. Cells were recorded in the presence of cycloheximide for 1 hour at 37°C and 5% CO_2._ Images were taken every minute. The direction of 3 PLP-eGFP-containing vesicles in HepG2-PLP-MAL cells was tracked for 6 min each in Fiji using the manual tracker plugin. The video is played at 3 frames/sec; total time for shown image sequence is 11 min.
(AVI)

## References

[1] Simons M, Nave K-A. Oligodendrocytes: Myelination and axonal support. Cold Spring Harb Perspect Biol. 2015;8(1): pii: a020479 10.1101/cshperspect.a020479 26101081 PMC4691794

[2] de Vries H, Hoekstra D. On the biogenesis of the myelin sheath: cognate polarized trafficking pathways in oligodendrocytes. Glycoconj J. 2000;17: 181–190. 11201789 10.1023/a:1026533021994

[3] Gielen E, Baron W, Vandeven M, Steels P, Hoekstra D, Ameloot M. Rafts in oligodendrocytes: evidence and structure-function relationship. Glia. 2006;54: 499–512. 16927294 10.1002/glia.20406

[4] Baron W, Hoekstra D. On the biogenesis of myelin membranes: Sorting, trafficking and cell polarity. FEBS Lett. 2010;584: 1760–70. 10.1016/j.febslet.2009.10.085 19896485

[5] White R, Krämer-Albers E-M. Axon-glia interaction and membrane traffic in myelin formation. Front Cell Neurosci. 2014;7: 284 10.3389/fncel.2013.00284 24431989 PMC3880936

[6] Simons M, Snaidero N, Aggarwal S. Cell polarity in myelinating glia: from membrane flow to diffusion barriers. Biochim Biophys Acta. 2012;1821: 1146–53. 10.1016/j.bbalip.2012.01.011 22314181

[7] Low SH, Chapin SJ, Weimbs T, Kömüves LG, Bennett MK, Mostov KE. Differential localization of syntaxin isoforms in polarized Madin-Darby canine kidney cells. Mol Biol Cell. 1996;7: 2007–2018. 8970161 10.1091/mbc.7.12.2007 PMC276046

[8] Fujita H, Tuma PL, Finnegan CM, Locco L, Hubbard AL. Endogenous syntaxins 2, 3 and 4 exhibit distinct but overlapping patterns of expression at the hepatocyte plasma membrane. Biochem J. 1998;329: 527–38. 9445379 10.1042/bj3290527 PMC1219073

[9] Bijlard M, Klunder B, de Jonge JC, Nomden A, Tyagi S, de Vries H, et al Transcriptional expression of myelin basic protein in oligodendrocytes depends on functional syntaxin 4: a potential correlation with autocrine signaling. Mol Cell Biol. 2015;35: 675–87. 10.1128/MCB.01389-14 25512606 PMC4301726

[10] Bello-Morales R, Pérez-Hernández M, Rejas MT, Matesanz F, Alcina A, López-Guerrero JA. Interaction of PLP with GFP-MAL2 in the human oligodendroglial cell line HOG. PLoS One. 2011;6: e19388 10.1371/journal.pone.0019388 21573057 PMC3090389

[11] Baron W, Ozgen H, Klunder B, de Jonge JC, Nomden A, Plat A, et al The major myelin-resident protein PLP is transported to myelin membranes via a transcytotic mechanism: involvement of sulfatide. Mol Cell Biol. 2015;35: 288–302. 10.1128/MCB.00848-14 25368380 PMC4295386

[12] Jahn O, Tenzer S, Werner HB. Myelin proteomics: molecular anatomy of an insulating sheath. Mol Neurobiol. 2009;40: 55–72. 10.1007/s12035-009-8071-2 19452287 PMC2758371

[13] Boison D, Büssow H, D’Urso D, Müller H-W, Stoffel W. Adhesive properties of proteolipid protein are responsible for the compaction of CNS myelin sheaths. J Neurosci. 1995;15: 5502–13. 7543946 10.1523/JNEUROSCI.15-08-05502.1995 PMC6577650

[14] Bakhti M, Snaidero N, Schneider D, Aggarwal S, Möbius W, Janshoff A, et al Loss of electrostatic cell-surface repulsion mediates myelin membrane adhesion and compaction in the central nervous system. Proc Natl Acad Sci USA. 2013;110: 3143–8. 10.1073/pnas.1220104110 23382229 PMC3581913

[15] Simons M, Kramer E-M, Macchi P, Rathke-Hartlieb S, Trotter J, Nave K-A, et al Overexpression of the myelin proteolipid protein leads to accumulation of cholesterol and proteolipid protein in endosomes/lysosomes: implications for Pelizaeus-Merzbacher disease. J Cell Biol. 2002;157: 327–36. 11956232 10.1083/jcb.200110138 PMC2199249

[16] Winterstein C, Trotter J, Krämer-Albers E-M. Distinct endocytic recycling of myelin proteins promotes oligodendroglial membrane remodeling. J Cell Sci. 2008;121: 834–42. 10.1242/jcs.022731 18303048

[17] Krämer-Albers E-M, Gehrig-Burger K, Thiele C, Trotter J, Nave K-A. Perturbed interactions of mutant proteolipid protein/DM20 with cholesterol and lipid rafts in oligodendroglia: implications for dysmyelination in spastic paraplegia. J Neurosci. 2006;26: 11743–52. 17093095 10.1523/JNEUROSCI.3581-06.2006 PMC6674790

[18] Feldmann A, Amphornrat J, Schönherr M, Winterstein C, Möbius W, Ruhwedel T, et al Transport of the major myelin proteolipid protein is directed by VAMP3 and VAMP7. J Neurosci. 2011;31: 5659–72. 10.1523/JNEUROSCI.6638-10.2011 21490207 PMC6622839

[19] Trajkovic K, Dhaunchak AS, Goncalves JT, Wenzel D, Schneider A, Bunt G, et al Neuron to glia signaling triggers myelin membrane exocytosis from endosomal storage sites. J Cell Biol. 2006;172: 937–48. 16520383 10.1083/jcb.200509022 PMC2063736

[20] Simons M, Krämer E-M, Thiele C, Stoffel W, Trotter J. Assembly of myelin by association of proteolipid protein with cholesterol- and galactosylceramide-rich membrane domains. J Cell Biol. 2000;151: 143–54. 11018060 10.1083/jcb.151.1.143 PMC2189802

[21] Kim T, Fiedler K, Madison DL, Krueger WH, Pfeiffer SE. Cloning and characterization of MVP17: a developmentally regulated myelin protein in oligodendrocytes. J Neurosci Res. 1995;42: 413–22. 8583510 10.1002/jnr.490420316

[22] Schaeren-Wiemers N, Valenzuela DM, Frank M, Schwab ME. Characterization of a rat gene, rMAL, encoding a protein with four hydrophobic domains in central and peripheral myelin. J Neurosci. 1995;15: 5753–64. 7643216 10.1523/JNEUROSCI.15-08-05753.1995 PMC6577631

[23] Frank M, Schaeren-Wiemers N, Schneider R, Schwab ME. Developmental Expression Pattern of the Myelin ProteolipiMAL Indicates Different Functions of MAL for Immature Schwann Cells and in a Late Step of CNS Myelinogenesis. J Neurochem. 2003;73: 587–597. 10.1046/j.1471-4159.1999.0730587.x 10428054

[24] Cheong KH, Zacchetti D, Schneeberger EE, Simons K. VIP17/MAL, a lipid raft-associated protein, is involved in apical transport in MDCK cells. Proc Natl Acad Sci USA. 1999;96: 6241–8. 10339572 10.1073/pnas.96.11.6241 PMC26866

[25] Zacchetti D, Peränen J, Murata M, Fiedler K, Simons K. VIP17/MAL, a proteolipid in apical transport vesicles. FEBS Lett. 1995;377: 465–9. d 8549777 10.1016/0014-5793(95)01396-2

[26] Ramnarayanan SP, Cheng CA, Bastaki M, Tuma PL. Exogenous MAL reroutes selected hepatic apical proteins into the direct pathway in WIF-B cells. Mol Biol Cell. 2007;18: 2707–15. 17494867 10.1091/mbc.E07-02-0096 PMC1924826

[27] Ozgen H, Schrimpf W, Hendrix J, de Jonge JC, Lamb DC, Hoekstra D, et al The lateral membrane organization and dynamics of myelin proteins PLP and MBP are dictated by distinct galactolipids and the extracellular matrix. PLoS One. 2014;9: e101834 10.1371/journal.pone.0101834 25003183 PMC4086962

[28] Frank M, van der Haar ME, Schaeren-Wiemers N, Schwab ME. rMAL is a glycosphingolipid-associated protein of myelin and apical membranes of epithelial cells in kidney and stomach. J Neurosci. 1998;18: 4901–13. 9634556 10.1523/JNEUROSCI.18-13-04901.1998 PMC6792556

[29] Schaeren-Wiemers N, Bonnet A, Erb M, Erne B, Bartsch U, Kern F, et al The raft-associated protein MAL is required for maintenance of proper axon-glia interactions in the central nervous system. J Cell Biol. 2004;166: 731–42. 15337780 10.1083/jcb.200406092 PMC2172435

[30] Frank M, Atanasoski S, Sancho S, Magyar JP, Rülicke T, Schwab ME, et al Progressive segregation of unmyelinated axons in peripheral nerves, myelin alterations in the CNS, and cyst formation in the kidneys of myelin and lymphocyte protein-overexpressing mice. J Neurochem. 2000;75: 1927–39. 11032882 10.1046/j.1471-4159.2000.0751927.x

[31] Maier O, van der Heide T, van Dam A-M, Baron W, De Vries H, Hoekstra D. Alteration of the extracellular matrix interferes with raft association of neurofascin in oligodendrocytes. Potential significance for multiple sclerosis? Mol Cell Neurosci. 2005;28: 390–401. 15691718 10.1016/j.mcn.2004.09.012

[32] Bsibsi M, Nomden A, van Noort JM, Baron W. Toll-like receptors 2 and 3 agonists differentially affect oligodendrocyte survival, differentiation, and myelin membrane formation. J Neurosci Res. 2012;90: 388–398. 10.1002/jnr.22767 21971760

[33] Richter-Landsberg C, Heinrich M. OLN-93: a new permanent oligodendroglia cell line derived from primary rat brain glial cultures. J Neurosci Res. 1996;45: 161–73. 8843033 10.1002/(SICI)1097-4547(19960715)45:2<161::AID-JNR8>3.0.CO;2-8

[34] Baron W, Bijlard M, Nomden A, de Jonge JC, Teunissen CE, Hoekstra D. Sulfatide-mediated control of extracellular matrix-dependent oligodendrocyte maturation. Glia. 2014;62: 927–42. d 10.1002/glia.22650 24578319

[35] de Vries H, Schrage C, Hoekstra D. An apical-type trafficking pathway is present in cultured oligodendrocytes but the sphingolipid-enriched myelin membrane is the target of a basolateral-type pathway. Mol Biol Cell. 1998;9: 599–609. 9487129 10.1091/mbc.9.3.599 PMC25288

[36] Ranscht B, Clapshaw PA, Price J, Noble M, Seifert W. Development of oligodendrocytes and Schwann cells studied with a monoclonal antibody against galactocerebroside. Proc Natl Acad Sci USA. 1982;79: 2709–13. 7045870 10.1073/pnas.79.8.2709 PMC346271

[37] Jung M, Sommer I, Schachner M, Nave KA. Monoclonal antibody O10 defines a conformationally sensitive cell-surface epitope of proteolipid protein (PLP): evidence that PLP misfolding underlies dysmyelination in mutant mice. J Neurosci. 1996;16: 7920–9. 8987820 10.1523/JNEUROSCI.16-24-07920.1996 PMC6579218

[38] Greenfield EA, Reddy J, Lees A, Dyer CA, Koul O, Nguyen K, et al Monoclonal antibodies to distinct regions of human myelin proteolipid protein simultaneously recognize central nervous system myelin and neurons of many vertebrate species. J Neurosci Res. 2006;83: 415–31. 16416423 10.1002/jnr.20748

[39] Schindelin J, Arganda-Carreras I, Frise E, Kaynig V, Longair M, Pietzsch T, et al Fiji: an open-source platform for biological-image analysis. Nat Methods. 2012;9: 676–82. 10.1038/nmeth.2019 22743772 PMC3855844

[40] Martín-Belmonte F, Kremer L, Albar JP, Marazuela M, Alonso MA. Expression of the MAL gene in the thyroid: the MAL proteolipid, a component of glycolipid-enriched membranes, is apically distributed in thyroid follicles. Endocrinology. 1998;139: 2077–84. 9528996 10.1210/endo.139.4.5875

[41] Caduff J, Sansano S, Bonnet A, Suter U, Schaeren-Wiemers N. Characterization of GFP-MAL expression and incorporation in rafts. Microsc Res Tech. 2001;52: 645–55. 11276117 10.1002/jemt.1049

[42] Puertollano R, Alonso MA. MAL, an integral element of the apical sorting machinery, is an itinerant protein that cycles between the trans-Golgi network and the plasma membrane. Mol Biol Cell. 1999;10: 3435–47. 10512878 10.1091/mbc.10.10.3435 PMC25613

[43] White R, Gonsior C, Bauer NM, Krämer-Albers E-M, Luhmann HJ, Trotter J. Heterogeneous nuclear ribonucleoprotein (hnRNP) F is a novel component of oligodendroglial RNA transport granules contributing to regulation of myelin basic protein (MBP) synthesis. J Biol Chem. 2012;287: 1742–54. 10.1074/jbc.M111.235010 22128153 PMC3265857

[44] Müller C, Bauer NM, Schäfer I, White R. Making myelin basic protein -from mRNA transport to localized translation. Front Cell Neurosci. 2013;7: 169 10.3389/fncel.2013.00169 24098271 PMC3784684

[45] Trifilieff E. Synthesis and secondary structure of loop 4 of myelin proteolipid protein: effect of a point mutation found in Pelizaeus-Merzbacher disease. J Pept Res. 2005;66: 101–10. 16083437 10.1111/j.1399-3011.2005.00278.x

[46] van IJzendoorn SC, Hoekstra D. (Glyco)sphingolipids are sorted in sub-apical compartments in HepG2 cells: a role for non-Golgi-related intracellular sites in the polarized distribution of (glyco)sphingolipids. J Cell Biol. 1998;142: 683–96. 9700158 10.1083/jcb.142.3.683 PMC2148170

[47] Simons K, Ikonen E. Functional rafts in cell membranes. Nature. 1997;387: 569–72. 9177342 10.1038/42408

[48] Taylor CM, Coetzee T, Pfeiffer SE. Detergent-insoluble glycosphingolipid/cholesterol microdomains of the myelin membrane. J Neurochem. 2002;81: 993–1004. 12065611 10.1046/j.1471-4159.2002.00884.x

[49] Frank M, Schaeren-Wiemers N, Schneider R, Schwab ME. Developmental expression pattern of the myelin proteolipid MAL indicates different functions of MAL for immature Schwann cells and in a late step of CNS myelinogenesis. J Neurochem. 1999;73: 587–97. 10428054 10.1046/j.1471-4159.1999.0730587.x

[50] Saravanan K, Schaeren-Wiemers N, Klein D, Sandhoff R, Schwarz A, Yaghootfam A, et al Specific downregulation and mistargeting of the lipid raft-associated protein MAL in a glycolipid storage disorder. Neurobiol Dis. 2004;16: 396–406. 15193296 10.1016/j.nbd.2004.03.008

[51] Werner HB, Krämer-Albers E-M, Strenzke N, Saher G, Tenzer S, Ohno-Iwashita Y, et al A critical role for the cholesterol-associated proteolipids PLP and M6B in myelination of the central nervous system. Glia. 2013; 61(4):567–86. 10.1002/glia.22456 23322581

[52] Arvanitis DN, Min W, Gong Y, Heng YM, Boggs JM. Two types of detergent-insoluble, glycosphingolipid/cholesterol-rich membrane domains from isolated myelin. J Neurochem. 2005;94: 1696–710. 16045452 10.1111/j.1471-4159.2005.03331.x

[53] Frank M. MAL, a proteolipid in glycosphingolipid enriched domains: functional implications in myelin and beyond. Prog Neurobiol. 2000;60: 531–44. 10739088 10.1016/s0301-0082(99)00039-8

[54] Magal LG, Yaffe Y, Shepshelovich J, Aranda JF, de Marco M del C, Gaus K, et al Clustering and lateral concentration of raft lipids by the MAL protein. Mol Biol Cell. 2009;20: 3751–62. 10.1091/mbc.E09-02-0142 19553470 PMC2777934

[55] Coetzee T, Suzuki K, Nave KA, Popko B. Myelination in the absence of galactolipids and proteolipid proteins. Mol Cell Neurosci. 1999;14: 41–51. 10433816 10.1006/mcne.1999.0768

[56] Aggarwal S, Yurlova L, Snaidero N, Reetz C, Frey S, Zimmermann J, et al A size barrier limits protein diffusion at the cell surface to generate lipid-rich myelin-membrane sheets. Dev Cell. 2011;21: 445–56. 10.1016/j.devcel.2011.08.001 21885353

