# Supplementary figures and images for: MAL Is a Regulator of the Recruitment of Myelin Protein PLP to Membrane Microdomains
Source: PLoS One. 2016 May 12;11(5):e0155317. doi: 10.1371/journal.pone.0155317 (PMC4865042; doi:10.1371/journal.pone.0155317)

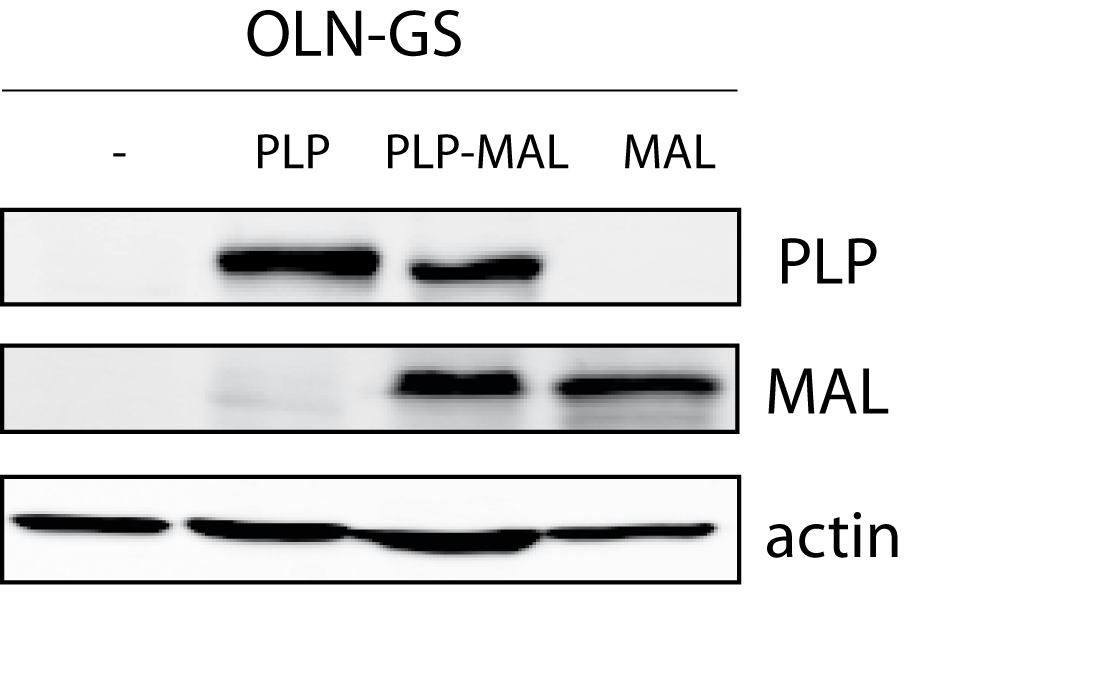

Supplement: S1 Fig — The expression of MAL and PLP in OLN-GS, OLN-PLP-GS, OLN-PLP-GS-MAL and OLN-GS-MAL cells were analyzed by Western blotting using anti-PLP (4C2) and anti-MAL (6D9) antibodies. Actin serves as a loading control. Note that OLN-GS do not express endogenous PLP and MAL, while the expression levels of PLP-eGFP and mCherry-MAL were similar in OLN-PLP-GS-MAL as compared to OLN-PLP-GS and OLN-GS-MAL cells respectively. (TIF) [file pone.0155317.s001.tif]
